# Stability and Activity of the Antimicrobial Peptide Leg1 in Solution and on Meat and Its Optimized Generation from Chickpea Storage Protein

**DOI:** 10.3390/foods10061192

**Published:** 2021-05-25

**Authors:** Marie-Louise Heymich, Showmika Srirangan, Monika Pischetsrieder

**Affiliations:** Chair of Food Chemistry, Department of Chemistry and Pharmacy, Friedrich-Alexander Universität Erlangen-Nürnberg (FAU), Nikolaus-Fiebiger-Str. 10, 91058 Erlangen, Germany; marie-louise.heymich@fau.de (M.-L.H.); showmika.srirangan@fau.de (S.S.)

**Keywords:** antimicrobial peptides, chickpea, chymotryptic hydrolysis, counter-ions, digestion optimization, Leg1, meat

## Abstract

The antimicrobial peptide Leg1 (RIKTVTSFDLPALRFLKL) from chickpea legumin is active against spoilage bacteria, yeast, and mold. The present study tested its effectiveness under food storage conditions and examined options to obtain a food-grade agent. The minimum inhibitory concentration (MIC) of Leg1 against *E.* *coli* (62.5 µM) proved stable over seven days at 20 °C or 4 °C. It was not influenced by reduced pH (5.0 vs. 6.8), which is relevant in food such as meat. An incubation temperature of 20 °C vs. 37 °C reduced the MIC to 15.6/7.8 µM against *E.* *coli*/*B.* *subtilis*. With a minimum bactericidal concentration in meat of 125/15.6 µM against *E.* *coli/B.* *subtilis*, Leg1 is equivalently effective as nisin and 5000–82,000 times more active than sodium benzoate, potassium sorbate, or sodium nitrite. Replacing the counter-ion trifluoroacetate derived from peptide synthesis by the more natural alternatives acetate or chloride did not impair the activity of Leg1. As an alternative to chemical synthesis, an optimized protocol for chymotryptic hydrolysis was developed, increasing the yield from chickpea legumin by a factor of 30 compared to the standard procedure. The present results indicate that food-grade Leg1 could possibly be applicable for food preservation.

## 1. Introduction

Food-spoilage microorganisms are a major cause of food loss and waste. For example, about 20% of the global meat produce is lost mainly as a consequence of microbial contamination. Controlling the load of microorganisms in animal products is therefore an important approach for reducing food waste [1]. Antimicrobial peptides (AMPs) may evolve into an interesting new class of preservatives. They interact specifically with the bacterial membrane leading to membrane disintegration and, consequently, to bacterial death [2]. Due to this general mechanism, AMPs often show broad-spectrum activity against a wide range of bacteria including antibiotic-resistant strains [3]. In recent decades, an increasing number of antimicrobial peptides has been identified. According to the Antimicrobial Peptides Database (APD), 3257 AMPs have been listed from different sources including some synthetic peptides: 2414 are derived from animals, 360 from plants, 365 from bacteria, 22 from fungi, eight from protists, and five from archaea. As to the most prominent effects, 83.67% of all identified peptides have antibacterial, 37.16% antifungal, 20.77% anti-candida, 5.86% antiviral, and 4.16% antiparasitic bioactivities (https://wangapd3.com/main.php, accessed on 13 April 2021). To date, however, nisin (E 234) is the only peptide that has been approved as food preservative in the European Union (Annex II of Regulation (EC) 1333/2008). Nisin is produced by *Lactococcus lactis* and contains several unusual amino acids and thioether bridges. AMPs from food sources are of particular interest for food preservation, because they can be considered as food-grade, safe ingredients. To date, most food-derived AMPs have been identified in milk and milk products, where they are either naturally present or released from milk proteins by enzymatic hydrolysis or fermentation [4,5,6,7,8,9]. Additionally, other animal proteins and, in particular, plant foods can be interesting sources of bioactive peptides [10,11]. Recently, the antimicrobial peptide Leg1 (RIKTVTSFDLPALRFLKL) was identified in chymotrypsin hydrolysates from chickpea legumin [12]. The peptide is active against a range of food pathogens, spoilage bacteria, yeast, and mold [12,13]. Because of this broad activity and the peptide’s origin from a common food, Leg1 could become a potential food preservative.

One possible constraint for the application of antimicrobial peptides in food can be their limited chemical stability. Peptides can be cleaved by non-enzymatic hydrolysis and proteolytic degradation [14,15]. Both reaction types are dependent on environmental conditions, such as water activity, temperature, and pH value [16]. High non-enzymatic proteolysis rates are mainly observed at low pH and/or high temperatures and are, therefore, of limited relevance for food storage [17]. Proteolytic degradation, in contrast, is likely to occur during food processing and storage. For instance, several endogenous peptidases in meat and fish are able to hydrolyze internal (endopeptidases) or terminal peptide bonds (exopeptidases) [18]. Additionally, plant and bacterial proteases are used in the meat industry to improve tenderization, which may influence the activity of peptide preservatives [19]. Additionally, reactive amino acid side chains of the peptides can be modified during the storage of meat, e.g., by oxidation or glycation, which may result in changes of the peptide structure and function [20,21].

For further assessment of whether Leg1 is applicable as food preservative, the present study tested its stability in solution and its stability and activity in meat. An additional goal of our experiments was to optimize the yield of Leg1 and its variant Leg2 (RIKTVTSFDLPALRWLKL), which differs only in one amino acid, from chymotryptic hydrolysates of chickpea.

## 2. Materials and Methods

### 2.1. Peptide Synthesis

The peptides Leg1 (RIKTVTSFDLPALRFLKL), Leg2 (RIKTVTSFDLPALRWLKL), and QILQWQ were commercially synthesized by ChinaPeptides Co. (Shanghai, China) using solid phase synthesis and delivered both as trifluoroacetate (TFA^−^) and acetate salt lyophilized powders (≥95%). The peptides were stored at −20 °C and dissolved in sterile water at a concentration of 2 mM. The stock solutions were diluted to the given concentrations with sterile water prior to use. Purity, mass, and sequence of the peptides were verified by enhanced mass spectrum full scan and enhanced product ion (EPI) scan measurements with a UHPLC system (Ultimate 3000 RS, Thermo Fisher, Idstein, Germany) coupled to a TripleQuad 6500^+^ mass spectrometer (AB Sciex, Darmstadt, Germany) equipped with Duospray Ion Source [12].

### 2.2. Bacterial Strains and Culture Conditions

*Escherichia (E.) coli* NEB 5α was obtained from New England Biolabs (Ipswich, MA, USA) and *Bacillus (B.) subtilis* ATCC 6051 from the Leibniz Institute DSZM-German Collection of Microorganisms and Cell Cultures (Braunschweig, Germany). Both bacterial strains were cultured in nutrient broth (8 g/L, Carl Roth, Karlsruhe, Germany) at 37 °C for 24 h under shaking in a temperature-controlled incubator shaker (Innova42R New Brunswick, New York, NY, USA) as described before [12].

### 2.3. Stability Test in Solution

Aqueous solutions containing 31.3 µM, 62.5 µM, 125 µM, 250 µM, and 500 µM of Leg1/Leg2 were stored in triplicates for 0, 1, 3, 7, 14, 21, and 28 days at 4 °C. In a second test set, the analogous storage experiment was carried out at room temperature (20 °C). Afterwards, the residual antimicrobial activity of the peptides was analyzed by a colorimetric microdilution assay containing peptide solution, *E. coli* suspension (1–5 × 10^6^ cfu/mL) and resazurin (6 mg/mL). After incubation for 16 h, the 96-well plate was measured at 570 nm [12]. For comparison, the stability of the approved preservatives nisin from *Lactococcus lactis* (62.5–1000 µM, Sigma-Aldrich, Taufkirchen, Germany), sodium benzoate (20,000–640,000 µM, ≥99.5%, Sigma-Aldrich), potassium sorbate (10,000–320,000 µM, 99%, Fisher Scientific, Schwerte, Germany), and sodium nitrite (5000–160,000 µM, ≥99%, Sigma-Aldrich) were dissolved in sterile water and tested in the same way. The antimicrobial activity was determined in triplicates and expressed by the minimal inhibitory concentration (MIC). The MIC was defined as the lowest concentration inhibiting bacterial growth. Ampicillin (25 µM; ampicillin sodium salt, Carl Roth) was used as positive and water as negative control.

To exclude possible interference with the resazurin assay, e.g., by antioxidative activity of the test substances, the antimicrobial activity of the peptides has been verified by a second independent microdilution assay recording bacterial growth at 600 nm [12].

### 2.4. Antimicrobial Assays at pH 5.0, pH 6.8, 20 °C, and 37 °C

The antimicrobial activity of Leg1 and Leg2 was tested at different pH (6.8 and 5.0) and temperatures (37 °C and 20 °C) in the resazurin assay as described above. While pH 6.8 was used according to the previously established standard protocol [12], pH 5.0 was the lowest pH at which bacterial growth could be observed. It was adjusted using 1 N HCl in all solutions including nutrient broth, water, and peptide solutions. Additionally, the resazurin assay was performed at 37 °C providing optimal bacterial growth and at 20 °C, which was the lowest temperature at which any growth was observed. Leg1 and Leg2 were tested in a concentration range between 0.5 µM and 125 µM in two-fold dilutions. For comparison, the approved preservatives nisin (0.5–250 µM), sodium benzoate (5000–80,000 µM), potassium sorbate (5000–160,000 µM), and sodium nitrite (2500–80,000 µM) were tested in the same way. Ampicillin (25 µM) was used as positive and sterile water as negative control. The MIC values were determined in triplicate.

### 2.5. Stability of Leg1 on Meat

To test the activity of Leg1 in a meat matrix and its stability during the storage of meat, pork from a local supermarket was cut into pieces (approx. 2 × 2 cm²). Four pieces were put into a petri dish and used immediately for further experiments. The petri dishes were prepared in triplicate. First, the meat was covered with the test solutions (10 µL each) in different concentrations. For comparison, the approved preservatives nisin, sodium benzoate, potassium sorbate, and sodium nitrite were tested in the same way as Leg1. After 2 min, the bacterial suspension (1–5 × 10^5^ cfu/mL) was pipetted onto the meat. After another 2 min at 20 °C, the meat was incubated at 37 °C for 16 h and then swabbed by sterile compact dry swabs, which were transferred into nutrient broth according to the manufacturer’s instruction (Carl Roth). The swabs were then transferred to agar plates, which were incubated at 37 °C until bacterial colonies were visible. When no growth was detectable on the agar plates after 16 h of incubation, the effect was interpreted as bactericidal activity, which was expressed as the minimal bactericidal concentration (MBC) on meat. The MBCs determined by Heymich et al., in solution [12] and at higher concentrations, were tested using twofold dilution steps. Thus, concentrations of 62.5–500 µM Leg1, 62.5–500 µM nisin, 320,000–1,280,000 µM sodium benzoate, 320,000–1,280,000 µM potassium sorbate, and 160,000–1,280,000 µM sodium nitrite were used against *E. coli*. The tests against *B. subtilis* were carried out with 7.8–500 µM Leg1, 7.8–500 µM nisin, 160,000–1,280,000 µM sodium benzoate, 640,000–1,280,000 µM potassium sorbate, and 80,000–1,280,000 µM sodium nitrite. Ampicillin (25 µM) was used as positive and water as negative control. The bactericidal activity on meat was analyzed in triplicate.

### 2.6. Antimicrobial Activity of Leg1 Using Different Counter-Ions

The antimicrobial activity of Leg1 bound to three different counter-ions, TFA^−^, acetate and chloride, was tested against *E. coli* and *B. subtilis*. The TFA^−^ salt and the acetate salt of Leg1 were obtained from ChinaPeptides Co. The chloride salt was generated by exchanging the TFA^−^ counter-ion in aqueous solution according to Sikora et al. [22]. The exchange was performed in 0.1 M HCl solution. The procedure including dissolution in HCl, incubation for 5 min and lyophilization was repeated four times. The peptide solutions and dilutions were prepared as described in Section 2.1. The antimicrobial activity of the peptide salts was tested using two antimicrobial assays, the inhibition curve assay and the resazurin assay according to Heymich et al. [12] at concentration ranges from 7.8 µM to 1000 µM. Ampicillin (25 µM) was used as positive and water as negative control. Briefly, 50 µL of the bacterial solution (1–5 × 10^5^ cfu/mL) was added to the test solution and the OD at 600 nm was recorded. The resazurin assay was carried out as described in Section 2.3. Additionally, the resazurin assay was performed for sodium trifluoroacetate, sodium chloride, and sodium acetate using the same concentration ranges. All MIC values of both assays were determined in triplicates.

### 2.7. Relative Quantification of Leg1 and Leg2 by UHPLC–MS/MS-sMRM

Relative quantification of Leg1 and Leg2 was achieved using a UHPLC system Ultimate 3000 RS coupled to a TripleQuad 6500^+^ mass spectrometer equipped with a Duospray Ion Source. For liquid chromatography, a C18 column (YMC Triart; 500 µm × 100 mm, 3 μm, 12 nm, 1/16”) was used at 35 °C with a flow rate of 30 µL/min. The sample (5 µL) was injected in full loop and the cut-off valve was set to 2 min and 55 min. A gradient of 0.1% formic acid (FA) as eluent A and 0.1% FA in acetonitrile (ACN) containing 2% water as eluent B (−15 min 2% B, 5 min 2% B, 55 min 43.4% B, 55.5 min 97% B, and 65 min 97% B) was used to separate the peptides. The Turbo Spray Ion Source was operated at 350 °C with 5000 V. The curtain gas was set to 40 psig, nebulizer gas to 40 psig, heating gas to 60 psi, de-clustering potential to 80 V, ion release delay to 30 V, and ion release width to 15 V. The detection window for the scheduled multiple reaction monitoring (sMRM) method was set to 300 s and Q1 mass (Da), Q3 mass (Da), retention time (min), and collision energy (V) were defined for every transition. The software Skyline 20.1.0.76 (MacCoss Lab) was used to create sMRM methods including precursor ions charged 1 to 4 and transitions charged 1 to 4 containing b/y ion types. The mass range was set between 200 *m/z* and 1500 *m/z* with a mass tolerance of 0.055 *m/z*. The milk peptide QILQWQ, which is not present in chickpea, was used as internal standard. To identify the quantifier and two qualifiers for the peptides, direct infusion, EPI scans and sMRM measurements of the synthesized peptides were performed. The data were evaluated using the software Analyst (version 1.6.3, AB Sciex, Darmstadt, Germany) and Skyline. The final parameters of the method are shown in Table 1. Representative sMRM chromatograms are included in the Supplementary Materials, Figure S1).

### 2.8. Optimization of Digestion Parameters

To maximize the yield of Leg1 and Leg2 released from the chickpea globulin fraction by chymotryptic hydrolysis, the digestion parameters enzyme/protein ratio, incubation time, temperature, protein pretreatment (denaturation/reduction/alkylation), and digestion stop were optimized in respect to the previously developed standard protocol [12]. The standard protocol included an enzyme/protein ratio of 1:50, 16 h of incubation at 25 °C, stopping of the digestion with FA, pretreatment by denaturation with urea, reduction of the disulfide bridges with dithiothreitol (DTT), and alkylation using iodoacetamide (IAA). Presently, each digestion parameter was modified separately while maintaining the other parameters identical to the standard protocol. Enzyme/protein ratios of 1:15, 1:50, and 1:100, digestion times of 4, 16, and 24 h, and temperatures of 25 °C and 37 °C were compared with regard to the yield of Leg1 and Leg2. The digestion was either stopped with FA, at 95 °C, or not at all. The standard protocol was repeated using urea as denaturation agent, urea combined with DTT and IAA, digestion buffer, and digestion buffer combined with DTT and IAA. The samples were analyzed by UHPLC–MS/MS-sMRM in triplicate. The standard peptide was added before MS measurement with a final concentration of 2.716 × 10^−4^ µg/µL. The peak areas were quantified with MultiQuant (AB Sciex); an index (analyte peak area/standard peak area) was created for each single measurement and each analyte. The results were expressed as percentages in comparison to the parameters used in the standard protocol [12], which were set to 100%. All experiments were performed in triplicate.

### 2.9. Statistical Analysis

The statistical analysis was conducted using the software GraphPad PRISM 8. For three points of comparison, one-way analysis of variance (ANOVA) with Turkey’s Honest Significant Different (HSD) test was used. For pairwise comparison (95% confidence interval) and for the comparison of two groups, a two-tailed, unpaired t-test was performed. The results are shown as the mean of triplicates ± standard deviation. The levels of significance were * *p* < 0.05 and ** *p* < 0.01.

## 3. Results and Discussion

The antimicrobial peptide Leg1 (RIKTVTSFDLPALRFLKL) derived from chickpea legumin is active against microorganisms, such as bacteria, yeast, and mold, which cause food spoilage or food infection [12,13]. To further assess whether Leg1 could be a potential food preservative, its stability was tested in solution and in a food matrix. Leg2 (RIKTVTSFDLPALRWLKL), which is also obtained from chickpea legumin, is a variant that differs only in one amino acid from Leg1 (F→W). The variation did not change the antimicrobial activity as demonstrated by identical MIC values of Leg1 and Leg2 against each of 16 previously tested bacterial strains [12]. Therefore, the present study focused on Leg1. However, some of the experiments were also carried out with Leg2.

### 3.1. Stability of Leg1 in Solution

After storing solutions of Leg1 for 0, 1, 3, 7, 14, 21, and 28 days at 20 °C or 4 °C, the antimicrobial activity was tested by the resazurin assay against *E. coli*. The MIC values were determined for each incubation time. At both temperatures, the MIC of 62.5 µM was stable during storage for up to seven days. Only after 14, 21, and 28 days did the MIC value increase to 125 µM (Table 2). For comparison, Leg2 and the approved preservatives nisin (125 µM), sodium benzoate (40,000 µM), potassium sorbate (20,000 µM), and sodium nitrite (10,000 µM) were tested in the same way. Leg2 showed the same behavior as Leg1, whereas the other preservatives were stable for the entire incubation time.

During short and long-term storage, proteinogenic amino acids may be modified by non-enzymatic reactions or proteolysis [23]. These reactions are strongly dependent on the amino acid composition [24]. In a pure aqueous solution, for example, oxidation of lysine or tryptophan or dehydration of serine or threonine may occur. Since Leg1/Leg2 remained stable for at least seven days, it can be concluded that modifications of reactive amino acids during storage at room temperature are not likely or that possible modifications do not affect the antimicrobial activity. Previous studies confirmed a high stability of antimicrobial peptides, even at higher temperatures. Baindara et al. observed that the antimicrobial activity of a peptide remained stable during incubation for 30 min at up to 100 °C, but reported significant reduction at 121 °C [25]. Stability up to 100 °C was also described for the peptide HKPLP by Sun et al. [26] and for 15 different peptides by Ebbensgaard et al. [27]. Additionally, Georgalaki et al. reported thermal stability of the food-grade antibiotic macedocin during short-term heating, long-term incubation for up to four weeks at 30 °C, and even full activity after autoclaving at 121 °C for 20 min [28].

The stability of peptides is dependent on the individual peptide sequence. The present results indicate that aqueous solutions of Leg1 can be stored before use for up to four weeks at room temperature or 4 °C while the MIC will only increase by one dilution step.

### 3.2. MIC of Leg1 Dependent on pH and Temperature

The antimicrobial activity of Leg1 was analyzed at pH 6.8/pH 5.0 and at 37 °C/20 °C to examine if differences in pH and temperature can affect the activity. In previous studies, the activity of Leg1/Leg2 was analyzed at 37 °C and pH 6.8, which are optimal growing conditions for the tested bacteria. However, food is usually stored at or below room temperature (20–22 °C) and the pH value in foods is often slightly acidic. In meat, pH values between 5 and 7 are common. Table 3 displays the results for the different incubation temperatures for Leg 1 and, for comparison, Leg2 and the approved preservatives nisin, sodium benzoate, potassium sorbate, and sodium nitrite. The MIC values of Leg1 against *E. coli* and *B. subtilis* decreased twofold at 20 °C compared to the temperature of 37 °C. Hence, for foods stored at or below room temperature lower peptide concentrations are necessary to inhibit bacterial growth. Lower MICs at 20 °C were also observed for Leg2 and the approved preservatives. The MIC of Leg2 against *B. subtilis* decreased fourfold to 4.0 µM. In our experimental setup, possible effects at temperatures below 20 °C could not be determined because no growth of *E. coli* and *B. subtilis* was detectable at lower temperatures, even in the absence of preservatives. Therefore, additional experiments are required to test the antimicrobial activity of Leg1 against psychrophilic bacteria at refrigeration temperatures.

Shifting the pH to 5.0 did not change the activity of Leg1 indicating that the peptide is also active at slightly acidic pH (Table 4). Similar results were obtained for Leg2 and nisin. The MIC values of the approved preservatives sodium benzoate and potassium sorbate were very high at pH 6.8 and did not change at pH 5.0. For both compounds, an activity optimum below pH 5.0 was reported [29]. The MIC of sodium nitrite at pH 5.0 was more than five times (*E. coli*) and 16 times (*B. subtilis*) lower compared to MICs under optimal growth conditions indicating its improved activity at pH 5.0. In the present experiments, the pH test range was also limited by the low growth rate of both test strains in a more acidic environment so that further experiments are required using acid-tolerant bacteria. 

Leg1, Leg2, and nisin are highly active in a pH range of 5.0–6.8. Sodium benzoate and potassium sorbate are usually applied at more acidic pH values below 5 and sodium nitrite at pH 5. Therefore, antimicrobial peptides could meet the demand for preservatives, applicable for neutral and slightly acidic foods. In particular, Leg 1 showed high activity in the pH range most relevant for meat (pH 5–7).

### 3.3. Bactericidal Activity of Leg1 on Meat

Leg1 proved to be stable during storage in aqueous solution. In contact with food, however, additional degradation reactions could occur, such as enzymatic proteolysis by food-derived proteases. Therefore, the bactericidal activity of the test substances was also assessed under food-storage conditions using routine test methods for foodstuffs. For this purpose, raw meat (pork) was pretreated with Leg1 and then inoculated with *E. coli* or *B. subtilis*. After 16 h at 37 °C, the bactericidal activity was measured. The MBCs of Leg1 on meat were 125 µM and 15.6 µM for *E. coli* and *B. subtilis*, respectively (Table 5), and thus equal to the MBCs observed in a previous microdilution assay [12]. This implies that meat constituents do not inhibit the activity of Leg1.

The MBCs of the approved preservatives nisin, sodium benzoate, potassium sorbate, and sodium nitrite were tested on meat in the same way. Nisin was equally effective as Leg1. The MBC values of sodium benzoate, potassium sorbate, and sodium nitrite on meat, however, ranged between 320,000 and 1,280,000 µM and exceeded the MBC in solution indicating a lower activity under the conditions of meat storage. Compared to Leg1, the MBCs of the conventional chemical preservatives were at least 2500 times higher. Despite the low pH value of meat (about 5.6) and the presence of proteases, Leg1 demonstrated high antimicrobial activity under the conditions of meat storage and clearly outperformed the efficacy of the approved chemical preservatives in this matrix.

### 3.4. Influence of the Counter-Ion on the Antimicrobial Activity of Leg1

**Figure 1:**
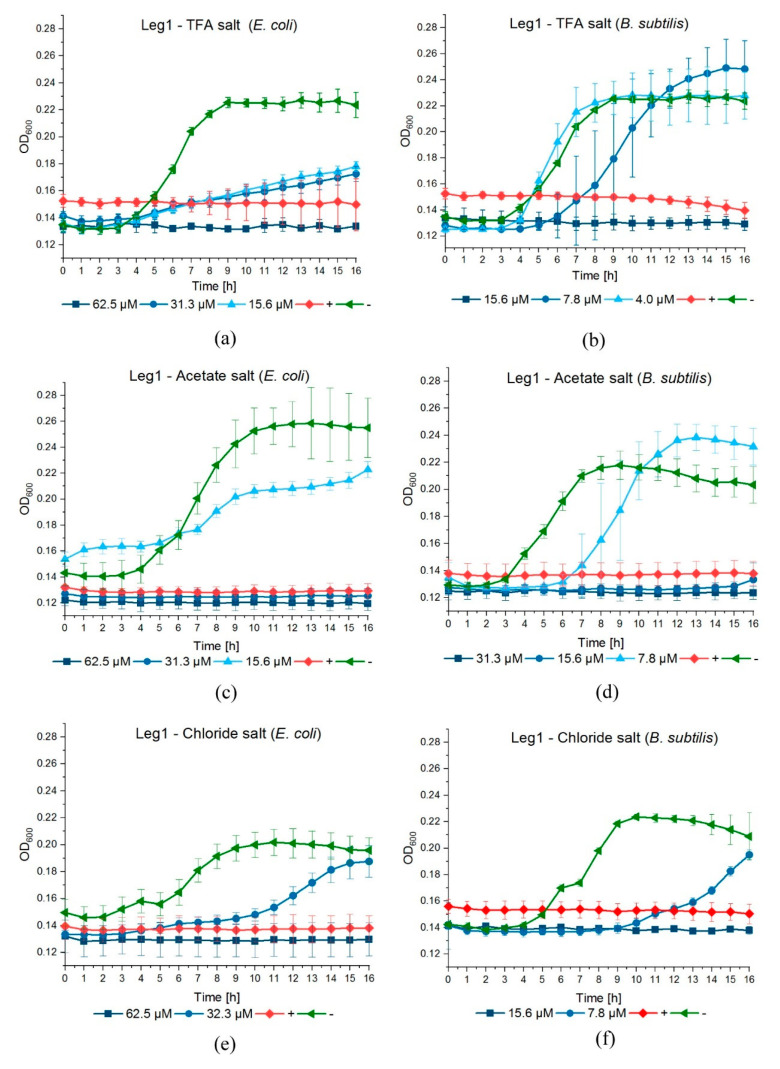
Inhibition curves of Leg1 against the growth of *E. coli* and *B. subtilis* over 16 h with different counter-ions: (**a**,**b**) trifluoroacetate (TFA^−^), (**c**,**d**) acetate, and (**e**,**f**) chloride. As controls, 25 µM ampicillin (positive control, +) and water (negative control, −) were used.

For the previous experiments, Leg1 was commercially synthesized as TFA^−^ salt. This is common in solid phase synthesis, because TFA is used for cleavage from the solid phase (resin) and is a component in the mobile phase for peptide purification by liquid chromatography. TFA is known to be cytotoxic by inhibiting cell proliferation and, therefore, could impact bacterial growth. Additionally, it could affect the secondary structure of peptides, which may be relevant for interaction with the bacterial membranes [30]. Since the unnatural counter-ion TFA^−^ is undesirable for the application in food, we assessed whether the observed antimicrobial activity of Leg1 relies on the counter-ion TFA^−^. For this purpose, Leg1 peptides prepared with the counter-ions TFA^−^, acetate, and chloride were subjected to the antimicrobial assay. Table 6 lists the MICs obtained in the resazurin assay and Figure 1 displays the bacterial growth curves. The MIC values were identical in the case of all three counter-ions, implying that TFA^−^ in the peptides has no significant impact on the antimicrobial strength and can be replaced by natural counter-ions. This is in accordance with the results of Greber et al. for short cationic lipopeptides [31]. In contrast, Sikora et al. described divergent MIC values of AMPs depending on their counter-ions and recommended the testing of peptides with different counter-ions individually [30]. Castiglia et al. did not observe any influence of acetate and chloride on MICs, but reported a better solubility of peptide chlorides [32].

In accordance with the present results, the sodium salts of TFA, acetate, and chloride did not show any antimicrobial effects in concentrations up to 1000 µM (Supplementary Materials, Table S1) confirming that the counter-ion has no influence on the antimicrobial activity of Leg1. Hence, the generation of Leg1 as food-grade chloride or acetate is possible without losing activity. The majority of peptide pharmaceuticals are manufactured in high quantities by chemical synthesis. This method is preferably employed for small to medium-sized peptides [33].

### 3.5. Optimization of the Chymotryptic Release of Leg1 and Leg2 from Chickpea Storage Protein

**Figure 2:**
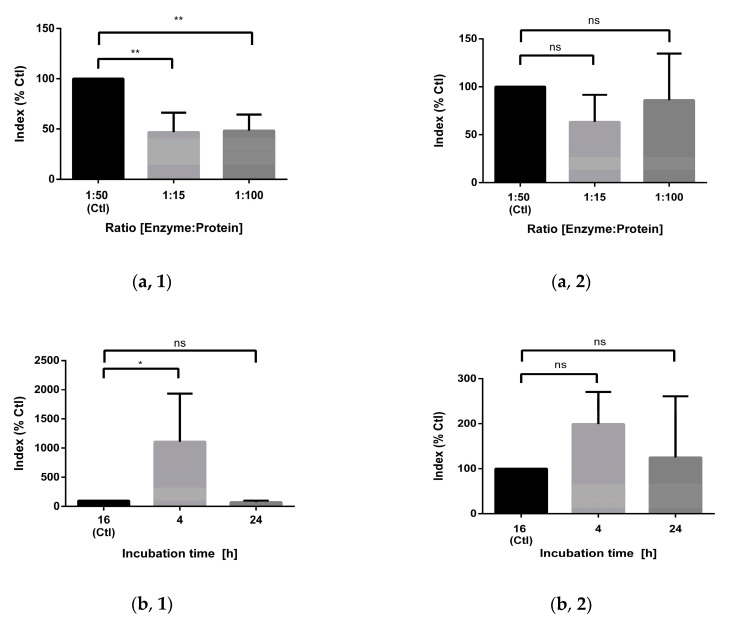

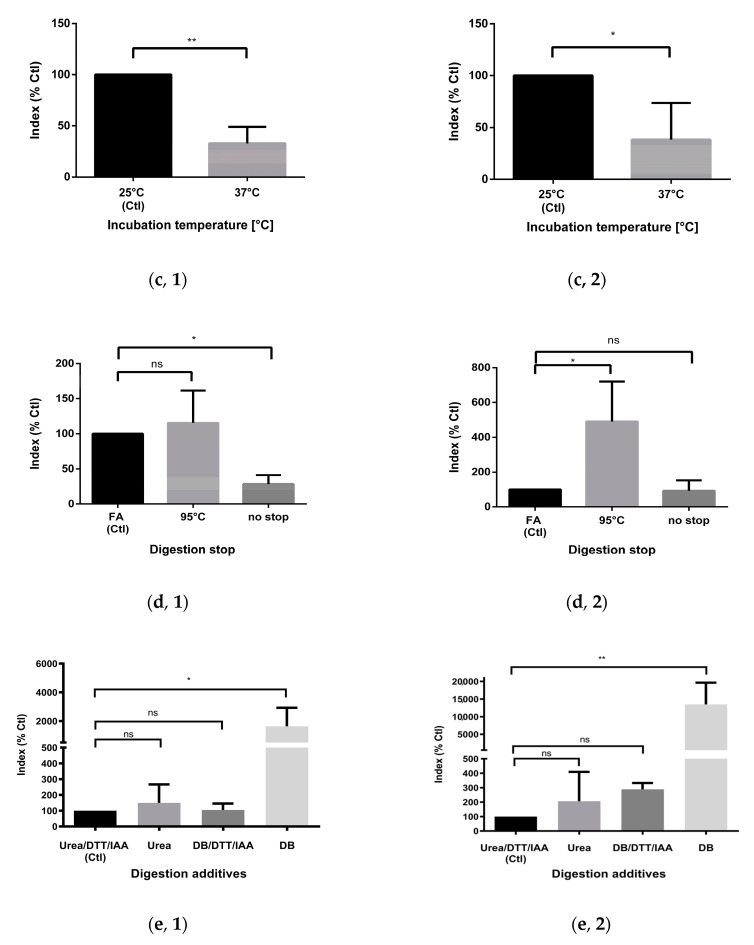

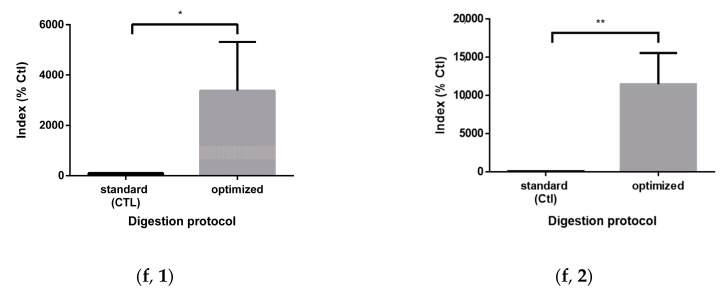
Optimization of the chymotryptic release of (left lane, 1) Leg1 and (right lane, 2) Leg2 from chickpea legumin. The following parameters were tested: (**a**) enzyme/protein ratio, (**b**) incubation time, (**c**) incubation temperature, (**d**) digestion stop (FA, formic acid), (**e**) digestion additives (DTT, dithiothreitol; IAA, iodoacetamide; DB, digestion buffer). (**f**) Comparison of the optimized digestion protocol with the standard protocol. The amounts of Leg1 and Leg2 were analyzed by ultrahigh performance-tandem mass spectrometry in scheduled multiple reaction monitoring mode (UHPLC–MS/MS-sMRM) and the results are shown as the index (analyte peak area/standard peak area) mean of triplicates ± standard deviation in percent compared to the standard protocol (Ctl). The levels of significance were ns, non-significant, * *p* < 0.05, ** *p* < 0.01.

As an alternative to synthesis, Leg1 could also be obtained from chickpea hydrolysates. The process should aim for a maximal yield avoiding, however, unnatural additives to improve the hydrolysis efficiency. Using chickpea protein as raw material, Leg1 and Leg2 are released in parallel from two different legumin variants. The present study optimized different parameters of the chymotryptic digestion such as enzyme/protein ratio, time, temperature, stopping of the digestion, and digestion pretreatments compared to the standard protocol [12]. The relative yields of Leg1 and Leg2 were analyzed by UHPLC–MS/MS in sMRM mode. The analytical parameters including precursor ions, quantifiers, qualifiers, retention times of Leg1, Leg2 and the internal standard are summarized in Table 1. In general, the results for Leg1 and Leg2 were very similar, but in some cases significant differences between the release behaviors of both peptides were observed (Figure 2). 

Besides the enzyme/protein ratio of 1:50 used in the previously developed standard protocol [12], higher (1:15) and lower (1:100) ratios were tested, but resulted in smaller or unchanged yields of Leg1 and Leg2. Reducing the incubation time (4 h vs. 16 h) increased the yield of Leg1 more than tenfold. The twofold increase observed for Leg2 release was, however, not significant. In contrast, extending the incubation time to 24 h did not change the yield. Both peptides possess missed cleavage sites at the C-termini of phenylalanine and tryptophan. In the case of Leg1, longer incubation times with chymotrypsin would therefore lead to the peptide fragments RIKTVTSF, DLPALRF, and LKL. Thus, it can be concluded that further degradation of the peptides Leg1/Leg2 during longer digestion periods overcompensates for their ongoing formation. Increasing the temperature from 25 °C to 37 °C reduced the release of Leg1 and Leg2. This is in accordance with the manufacturer`s guidelines for chymotrypsin. Its temperature optimum is 25 °C, in contrast to most proteases, which possess an optimal operation temperature of 37 °C.

In the standard protocol, the digestion stops after the addition of FA [12]. As an alternative, the hydrolysis could be terminated by enzyme inactivation at 95 °C. Furthermore, the necessity for stopping the hydrolysis was also examined. Interestingly, the termination of hydrolysis influenced the release of Leg1 and Leg2 differently. Whereas the heat impact considerably increased the yield of Leg2, no significant change was observed for Leg1 after heating. Unterminated hydrolysis, on the other hand, resulted in a lower yield of Leg1. This observation indicates that Leg1 and Leg2 may be further degraded during prolonged proteolysis. Despite the significant rise in generated Leg2 after short-term heating at 95 °C, stopping the digestion by FA was better reproducible, so that we did not change the standard protocol in this respect. Besides, high temperatures may promote non-enzymatic and thermal peptide degradation.

Prior to digestion, the standard protocol used for the comprehensive monitoring of the peptide profile in chickpea hydrolysates included reduction and alkylation steps to achieve high sequence coverage [12]. Protein denaturation by urea and DTT and further stabilization of the denatured protein by alkylation of reduced cysteine residues with IAA improves the accessibility of the proteins to chymotrypsin. In the course of varying the parameters to optimize peptide generation, chymotryptic hydrolysis was carried out without reduction/alkylation and without urea denaturation. In contrast to expectations, the yield of Leg1 and Leg2 increased by a factor of 20–100 when the protein extract was dissolved in plain digestion buffer. Thus, a simplified digestion protocol is suggested avoiding non-food grade additives. Instead of DTT, other reducing reagents can be used like tris-(2-chlorethylphosphate) and β-mercapto-ethanol. Additionally, the alkylation agent IAA can be replaced, for example, by chloroacetamide, acrylamide, or iodoacetic acid. Müller & Winter tested different combinations of reduction and alkylation reagents. Combined DTT/acrylamide resulted in the highest number of identified unique peptide sequences, whereas DTT/IAA led to the same yield as a protocol without reduction and alkylation steps [34]. Urea is used to obtain high solubility, but is also known to induce chemical modifications [35]. It depends on the amino acid composition of the proteins and the target peptide whether these agents are necessary. For the generation of Leg1/Leg2 from the chickpea albumin and globulin fraction, denaturation, reduction, and alkylation are not necessary and even inhibit the release of the peptides. Most likely, the peptides’ increased accessibility after sample pretreatment results in the hydrolysis of missed cleavage sites in Leg1/Leg2.

In summary, the optimized protocol including a shorter digestion time of 4 h and the direct protein hydrolysis without pretreatment increased the yield of Leg1 (30-fold) and Leg2 (110-fold) significantly (Figure 2f). Using the human pancreatic digestion enzyme chymotrypsin without chemical hydrolysis enhancers such as DTT and IAA has the additional benefit of providing a food-grade product.

## 4. Conclusions

The present results indicate that the stability of Leg1 in solution and its activity at slightly acid pH and on meat warrant further experiments to test its applicability in food preservation. Additional studies are now required to extend the assessment to more food pathogens and spoilage bacteria. MIC values of 250 and 500 µM Leg1 were observed, for example, against two different strains of *Listeria monocytogenes*, which are of particular relevance for meat spoilage [12]. As a next step, the application of Leg1 to further food products and under various storage conditions must be tested. Food-grade production of Leg1 could be achieved by chemical synthesis or by the chymotryptic digestion of chickpea storage proteins. In addition to chemical synthesis or enzymatic hydrolysis of food proteins, biotechnological processes, which are often rather cost-effective, can also be considered for large-scale production [36,37].

## Figures and Tables

**Table 1:** Relative quantification of Leg1 and Leg2 in chickpea chymotryptic hydrolysates by ultrahigh performance-tandem mass spectrometry in scheduled multiple reaction monitoring mode (UHPLC–MS/MS-sMRM). The analytical parameters of the method are displayed. QILQWQ was used as internal standard. The net charge is given by superscript numbers (^1+,2+,3+,4+^).

Peptide Sequence	Theoretical Mass (Da)	RT ^1^ (min)	Precursor Ion (*m*/*z*)	Quantifier Ion (*m*/*z*)	Collision Energy (eV)	Confirmation Ions (*m*/*z*)	
RIKTVTSFDLPALRFLKL (Leg1)	2118.59	36.1	530.33^4+^	957.02^1+^	30.4	710.42^2+^	581.34^2+^
RIKTVTSFDLPALRWLKL (Leg2)	2157.62	36.7	540.08^4+^	729.93^2+^	27.0	686.41^2+^	780.45^2+^
QILQWQ	814.94	25.2	408.22^2+^	242.16^3+^	22.6	333.16^2+^	355.23^1+^

^1^ Retention time.

**Table 2:** Stability of Leg1 during storage at 20 °C for 0–28 days. The residual antimicrobial activity against *E. coli* was measured by the resazurin assay and is expressed as minimal inhibitory concentration (MIC). For comparison, the stability of the variant Leg2 and the approved preservatives nisin, sodium benzoate, potassium sorbate, and sodium nitrite were analyzed in the same way. The same results were obtained during storage at 4 °C for 0–28 days.

	MIC (µM) at Day(s)						
	0	1	3	7	14	21	28
Leg1	62.5	62.5	62.5	62.5	125	125	125
Leg2	62.5	62.5	62.5	62.5	125	125	125
Nisin	125	125	125	125	125	125	125
Sodium benzoate	40,000	40,000	40,000	40,000	40,000	40,000	40,000
Potassium sorbate	20,000	20,000	20,000	20,000	20,000	20,000	20,000
Sodium nitrite	10,000	10,000	10,000	10,000	10,000	10,000	10,000

**Table 3:** Antimicrobial activity of Leg1 against *E. coli* and *B. subtilis* at different temperatures expressed as minimal inhibitory concentration (MIC). For comparison, the variant Leg2 and the approved preservatives nisin, sodium benzoate, potassium sorbate, and sodium nitrite were tested in the same way.

	MIC in µM (mg/mL) at Temperatures			
	37 °C	20 °C	37 °C	20 °C
	*E. coli*		*B. subtilis*	
Leg1	62.5 (0.13)	15.6 (0.03)	15.6 (0.03)	7.8 (0.02)
Leg2	62.5 (0.13)	15.6 (0.03)	15.6 (0.03)	4.0 (0.01)
Nisin	125 (0.42)	15.6 (0.05)	7.81 (0.03)	1.0 (0.004)
Sodium benzoate	40,000 (5.8)	20,000 (2.9)	80,000 (11.5)	10,000 (1.5)
Potassium sorbate	20,000 (3.0)	20,000 (3.0)	160,000 (24.0)	20,000 (3.0)
Sodium nitrite	10,000 (0.68)	20,000 (1.36)	40,000 (2.76)	20,000 (1.36)

**Table 4:** Antimicrobial activity of Leg1 against *E. coli* and *B. subtilis* at different pH values expressed as minimal inhibitory concentration (MIC). For comparison, the variant Leg2 and the approved preservatives nisin, sodium benzoate, potassium sorbate, and sodium nitrite were tested in the same way.

	MIC in µM (mg/mL) at pH			
	6.8	5.0	6.8	5.0
	*E. coli*		*B. subtilis*	
Leg1	62.5 (0.13)	62.5 (0.13)	15.6 (0.03)	15.6 (0.03))
Leg2	62.5 (0.13)	62.5 (0.13)	15.6 (0.03)	15.6 (0.03)
Nisin	125 (0.42)	125 (0.42)	7.81 (0.03)	7.81 (0.03))
Sodium benzoate	40,000 (5.8)	40,000 (5.8))	40,000 (5.8)	40,000 (5.8)
Potassium sorbate	40,000 (6.0)	40,000 (6.0)	80,000 (12.0)	80,000 (12.0)
Sodium nitrite	40,000 (2.76)	<2500	40,000 (2.76)	<2500

**Table 5:** Bactericidal activity of Leg1 against *E. coli* and *B. subtilis* on meat expressed as minimal bactericidal concentration (MBC). For comparison, the approved preservatives nisin, sodium benzoate, potassium sorbate, and sodium nitrite were tested in the same way.

	MBC in µM (mg/mL)	
	*E. coli*	*B. subtilis*
Leg1	125 (0.26)	15.6 (0.03)
Nisin	125 (0.42)	15.6 (0.052)
Sodium benzoate	1,280,000 (184.0)	640,000 (92.0)
Potassium sorbate	640,000 (96.0)	1,280,000 (192.0)
Sodium nitrite	640,000 (44.10)	320,000 (22.08)

**Table 6:** Antimicrobial activity expressed as minimal inhibitory concentration (MIC) of Leg1 with trifluoroacetate (TFA^−^), acetate, and chloride as counter-ions against *E. coli* and *B. subtilis*.

Counter-Ion	MIC (µM)	
	*E. coli*	*B. subtilis*
TFA^−^	62.5	15.6
acetate	62.5	15.6
chloride	62.5	15.6

## Data Availability

Data available on request.

## Supplementary Materials

The following are available online at https://www.mdpi.com/article/10.3390/foods10061192/s1, Table S1: Antimicrobial activity of sodium TFA^−^, sodium acetate, and sodium chloride against *E. coli* and *B. subtilis* displayed as minimum inhibitory concentration (MIC) in µM; Figure S1: Relative quantification of Leg1 and Leg2 in chymotryptic hydrolysates of the chickpea globulin fraction by ultrahigh-performance liquid chromatography–tandem mass spectrometry in scheduled reaction monitoring mode (UHPLC–MS/MS-sMRM): (A) Representative Chromatogram of a hydrolysate (4 h incubation) and the three MRM transitions of (B) QILQWQ (internal standard peptide), (C) Leg1, and (D) Leg2 as described in Table 1.
