# Supplementary material for: Stability and Activity of the Antimicrobial Peptide Leg1 in Solution and on Meat and Its Optimized Generation from Chickpea Storage Protein
Source: Foods. 2021 May 25;10(6):1192. doi: 10.3390/foods10061192 (PMC8227015; doi:10.3390/foods10061192)
Supplement: Supplementary file 1 [file foods-10-01192-s001.zip › foods-1235495-supplementary.pdf]

Supplementary Material

Stability and Activity of the Antimicrobial Peptide Leg1 in Solution and on Meat and Its Optimized Generation from Chickpea Storage Protein

Marie-Louise Heymich, Showmika Srirangan and Monika Pischetsrieder *

[1] Chair of Food Chemistry, Department of Chemistry and Pharmacy, Friedrich-Alexander Universität Erlangen-Nürnberg (FAU), Nikolaus-Fiebiger-Str. 10, 91058 Erlangen, Germany; marie-louise.heymich@fau.de; showmika.srirangan@fau.de; monika.pischetsrieder@fau.de

* Correspondence: monika.pischetsrieder@fau.de; Tel.: 0049-9131-85-65592

Table S1. Antimicrobial activity of sodium trifluoroacetate, sodium acetate and sodium chloride against *E. coli* and *B. subtilis* displayed as minimum inhibitory concentration (MIC) in µM.

	**Counter-ion**	**MIC (µM)**	
		E. coli	***B. subtilis***
Sodium trifluoroacetate	TFA^-	> 1000	> 1000
Sodium acetate	CH_3OO^-	> 1000	> 1000
Sodium chloride	Cl^-	> 1000	> 1000

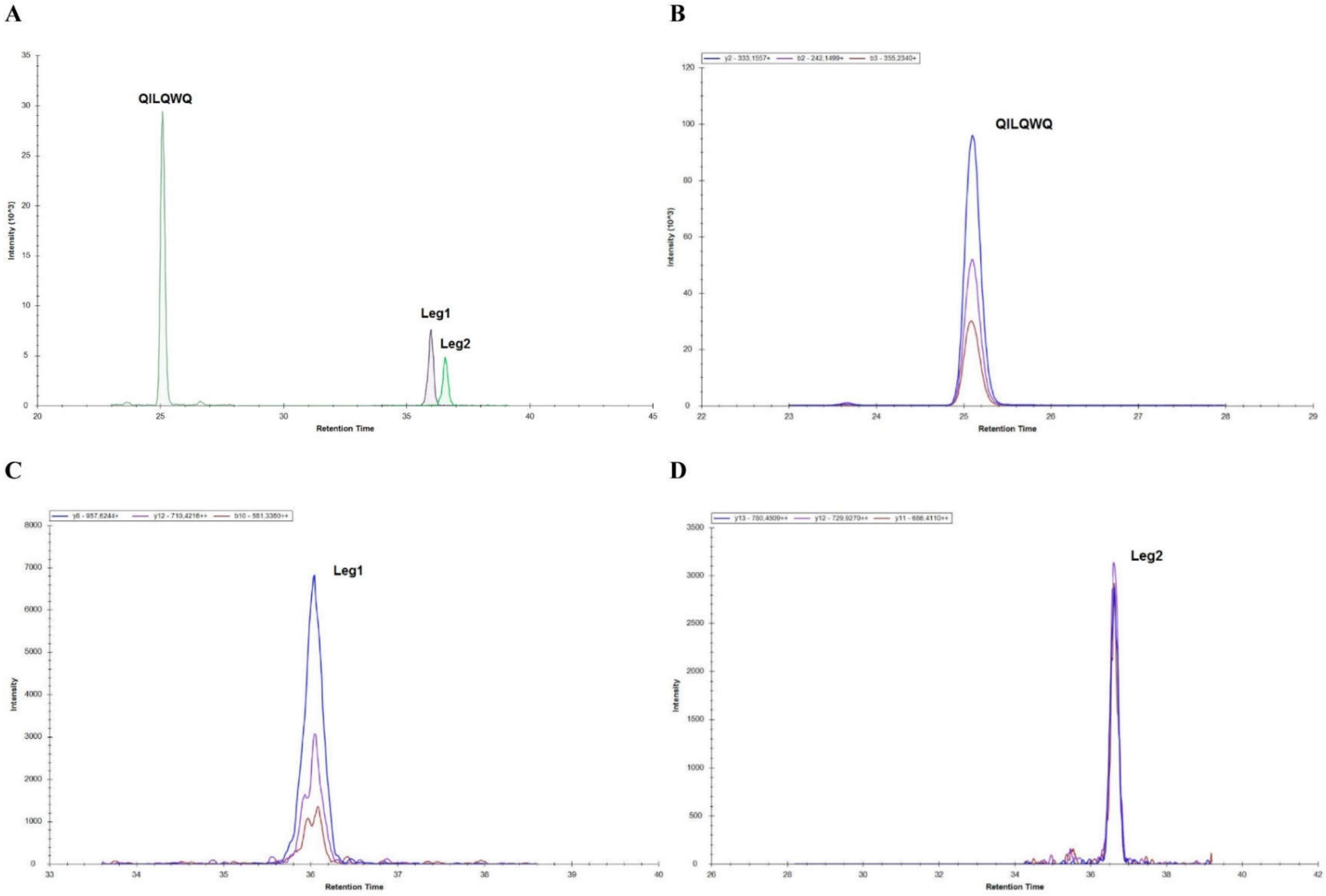

Figure S1. Relative quantification of Leg1 and Leg2 in chymotryptic hydrolysates of the chickpea globulin fraction by ultrahigh-performance liquid chromatography–tandem mass spectrometry in scheduled reaction monitoring mode (UHPLC–MS/MS-sMRM): A) Representative Chromatogram of a hydrolysate (4h incubation) and the three MRM transitions of B) QILQWQ (internal standard peptide), C) Leg1, and D) Leg2 as described in Table 1.
